# *Helicobacter pylori*-induced fibroblast-derived Serpin E1 promotes gastric cancer growth and peritoneal dissemination through p38 MAPK/VEGFA-mediated angiogenesis

**DOI:** 10.1186/s12935-023-03177-1

**Published:** 2023-12-16

**Authors:** Wei Cheng, Yonghui Liao, Yuan Xie, Qinrong Wang, Leilei Li, Yuanjia Chen, Yan Zhao, Jianjiang Zhou

**Affiliations:** 1 Key Laboratory of Endemic and Ethnic Diseases, Ministry of Education & Key Laboratory of Medical Molecular Biology of Guizhou Province, Guizhou Medical University, Guiyang, China; 2 Department of Hematology, Affiliated Hospital of Guizhou Medical University, Guiyang, China; 3 Prenatal Diagnosis Center of Qianxinan People’s Hospital, Xingyi, China

**Keywords:** Serpin E1, Fibroblasts, *Helicobacter pylori*, p38 MAPK, Angiogenesis

## Abstract

**Background:**

Fibroblasts, especially cancer-associated fibroblasts (CAFs), represent the predominant stromal cell population in the tumor microenvironment and have an important function in tumorigenesis by interacting with tumor cells. However, their interaction remains elusive in an inflammatory tumor microenvironment induced by *Helicobacter pylori* (*H. pylori*).

**Methods:**

The expression of Serpin family E member 1 (Serpin E1) was measured in fibroblasts with or without *H. pylori* infection, and primary gastric cancer (GC) cells. Serpin E1 knockdown and overexpression fibroblasts were generated using Serpin E1 siRNA or lentivirus carrying Serpin E1. Co-culture models of fibroblasts and GC cells or human umbilical vein endothelial cells (HUVECs) were established with direct contact or the Transwell system. In vitro functional experiments and in vivo tumorigenesis assay were employed to study the malignant behaviors of GC cells interacting with fibroblasts. ELISA was used for quantifying the levels of Serpin E1 and VEGFA in the culture supernatant. The tube formation capacity of HUVECs was assessed using a tube formation assay. Recombinant human Serpin E1 (recSerpin E1), anti-Serpin E1 antibody, and a MAPK pathway inhibitor were utilized to treat HUVECs for elucidating the underlying molecular mechanisms.

**Results:**

Serpin E1 was predominantly expressed in gastric CAFs. *H. pylori* infection significantly enhanced the expression and secretion of Serpin E1 by CAFs**.** Both fibroblast-derived Serpin E1 and recSerpin E1 enhanced the growth, invasion, and migration of GC cells, along with increased VEGFA expression and tube formation in HUVECs. Furthermore, the co-inoculation of GC cells and fibroblasts overexpressing Serpin E1 triggered the expression of Serpin E1 in cancer cells, which facilitated together xenograft tumor growth and peritoneal dissemination of GC cells in nude mice, with an increased expression of Ki67, Serpin E1, CD31 and/or VEGFA. These processes may be mediated by Serpin E1-induced migration and p38 MAPK/VEGFA-mediated angiogenesis of HUVECs.

**Conclusion:**

*H. pylori* infection induces Serpin E1 expression in fibroblasts, subsequently triggering its expression in GC cells through their interaction. Serpin E1 derived from these cells promotes the migration and p38 MAPK/VEGFA-mediated angiogenesis of HUVECs, thereby facilitating GC growth and peritoneal metastasis. Targeting Serpin E1 signaling is a potential therapy strategy for *H. pylori*-induced GC.

**Supplementary Information:**

The online version contains supplementary material available at 10.1186/s12935-023-03177-1.

## Background

Gastric cancer (GC) is one of the most common malignant diseases globally, with a fifth-place incidence and fourth-place mortality in 2021 [1]. The 5-year survival of GC remains below 25%, with lymph node metastasis occurring in over 10% of early-stage cases [2]. The pathogenesis of GC is complex and remains incompletely elucidated. Key factors influencing GC development include *Helicobacter pylori* (*H. pylori*) infection, tumor microenvironment (TME), host polymorphisms, and gene expression disorders. Of these, *H. pylori* infection is the strongest risk factor [3]. In 2018, *H. pylori* infection was estimated to be responsible for approximately 89% of new non-cardia GC cases and 20% of new cardia GC cases. [4]. The gastric mucosa of over half the world's population is colonized by *H. pylori*, which can cause chronic inflammation, ulcers, and even cancer if not completely eradicated [5].

Several studies have demonstrated a correlation between GC and *H. pylori* infection. For example, *H. pylori* induces genetic and epigenetic alterations via chronic inflammation, leading to GC development [6, 7]. *H. pylori* degrades tumor suppressor or activates oncogene to accelerate cell division and tumorigenic transformation and, ultimately, gastric carcinogenesis [8, 9]. Additionally, *H. pylori* disrupts cell tight junctions to induce epithelial-to-mesenchymal transition via downregulating adhesion molecules IQGAP1 and Afadin in gastric cells [10, 11]. *H. pylori* also promotes cancer cell stemness by activating the NF-кB, ERK, JNK, and Hippo pathways [12]. Despite all this, the molecular mechanism underlying *H. pylori*'s promotion of GC development remains incompletely understood. Consequently, researchers have recently focused on investigating the influence of *H. pylori* on TME in the stomach.

TME consists of diverse cellular components, including normal fibroblasts, cancer-associated fibroblasts (CAFs), immune cells, endothelial cells, etc., soluble molecules like chemokines, enzymes, cytokines, etc., and extracellular matrix (ECM). Notably, the interaction between fibroblasts and cancer cells not only promotes tumor development but also triggers the activation of fibroblasts [13]. The study by Krzysiek-Maczka and co-authors demonstrated that the infection with *H. pylori* induced the activation of gastric fibroblasts, leading to the induction of epithelial-to-mesenchymal transition in gastric RGM-1 cells through the secretion of transforming growth factor [14]. Furthermore, infection with *H. pylori* elevated the expression of VCAM1 in CAFs to promote GC cell invasion via the JAK/STAT1 pathway [15]. In our recent study [16], we reported that the association among *H. pylori*, normal fibroblasts, and GC cells induced the activation of fibroblast into CAFs and upregulation of Serpin family E member 1 (Serpin E1) by GC cells, thereby promoting gastric carcinogenesis. However, it remains unclear whether Serpin E1 is produced by fibroblasts or cancer cells in the inflammatory microenvironment induced by *H. pylori*, and the underlying mechanism by which Serpin E1 promotes GC development remains elusive.

In physiological conditions, Serpin E1 (also called PAI-1) is found in plasma at low concentrations, ranging from 5 to 50 ng/mL, in its active conformation. Conversely, it is predominantly retained in platelets at significantly higher levels (about 300 ng/mL) in its latent conformation, exhibiting only 2–5% functional activity [17]. Upon platelet activation, Serpin E1 was converted into active form and released into plasma to trigger the plasminogen/plasmin system through the inhibition of tissue- and urokinase-type plasminogen activators (uPA and tPA) [18]. As a direct inhibitor of tPA and uPA, Serpin E1 prevents plasminogen activation and fibrin clot degradation, thereby contributing to thrombosis development in pathological conditions associated with cardiovascular disease [19]. In addition, uPA-mediated plasminogen activation can induce pericellular proteolysis, tissue remodeling, and cell migration that favor tumor development [17]. Therefore, it is plausible that Serpin E1 may exert anti-tumor effects. However, recent studies find that Serpin E1 is overexpressed in certain forms of tumor and exhibits a positive correlation with tumor progression [20]. Growth factors, chemokines, and environmental stress, directly and indirectly, regulate Serpin E1 expression [21]. It should be noted that most of these findings were primarily derived from investigations involving epithelial cancer cells.

The present study revealed that Serpin E1 was primarily expressed in CAFs, and its expression and secretion were enhanced upon infection with *H. pylori*, which further induced the expression of Serpin E1 in GC cells through their interaction. Serpin E1 derived from these cells and recombinant human Serpin E1 (recSerpin E1, 140–04, PeproTech, USA) activated chemotactic migration and p38 mitogen-activated protein kinase (MAPK) / vascular endothelial growth factor (VEGF) A-mediated angiogenesis of endothelial cells, thereby promoting GC cell proliferation and peritoneal dissemination. Here, our results uncover a novel mechanism underlying the development and progression of *H. pylori*-induced GC.

## Materials and methods

### *H. pylori* strain and cell lines

The East Asian strain *H. pylori* GZ7 (*cagA* +) was previously isolated from a Chinese patient with GC [22]. The AGS human GC cell line (CRL-1739) and Hs738 human gastric fibroblast cell line (CRL-7869) were obtained from the American Type Culture Collection (ATCC, USA). Human GC cell line (MKN45) and the umbilical vein endothelial cells (HUVECs) were purchased from the Shanghai Cell Bank of the Chinese Academy of Sciences (Shanghai, China). Three primary CAFs and GC cells from the same patients with GC were previously isolated from resected GC tissues [16]. All cells and strains were tested to eliminate the possibility of mycoplasma contamination. Informed consent was obtained from all patients, and the research was approved by Guizhou Medical University (No. 2017(43)).

*H. pylori* was cultured on Columbia blood agar plates supplemented with 10% sheep blood and 100 U/ml of *H. pylori* selective supplement (Oxoid, Basingstoke, UK) in a microaerobic environment with a temperature of 37 °C. All cells were grown in DMEM (D6429, Sigma, USA) supplemented 10% fetal bovine serum (FBS, #16000–044, Gibco, USA) and 1% penicillin–streptomycin (SV30010, Hyclone, USA).

### Knockdown of Serpin E1

The siRNA targeting Serpin E1 (siSerpin E1) and control scrambled siRNA (siNC) were obtained from GenePharma Co., Ltd. (Shanghai, China). CAFs (2 × 10^5^ cells) were grown in a 6-well plate and transfected with siSerpin E1 and siNC with Lipofectamine 2000 (#11668019, Invitrogen, Waltham, MA). Forty-eight hours later, the cells were harvested for western blotting and RT-qPCR analysis to determine Serpin E1expression. The sequence for siSerpin E1 (5′–3′) was as follows: GCUCAGACCAACAAGUUCATT; while that of siNC (5′–3′) was: UUCUUCGAACGUGUCACG UTT.

### Construction of Serpin E1 lentivirus vector and generation of stable cell lines

Lentiviral vector expressing Serpin E1-enhanced green fluorescent protein (EGFP, non-fusion) and an EGFP-expressing control vector was constructed by JiKai Gene Company (Shanghai, China). Briefly, the human coding sequence (CDS) of Serpin E1(NM_000602) was obtained from the GenBank and synthesized with AgeI and NheI restriction enzyme sites at the two terminals. After restriction digestion with AgeI and NheI, the Serpin E1 CDS was ligated into the GV367 plasmid (Ubi-(AgeI)MCS(NheI)-SV40-EGFP-IRES-puromycin, Ji Kai), followed by transformation into E. coli DH5α. Subsequently, DH5α cells were cultured, and recombinant plasmid DNA was extracted for identification through sequencing. After successful construction of the recombinant GV367 lentiviral vector, GV367 and helper 1.0 and helper 2.0 (auxiliary packaging plasmids, obtained from JiKai Company) co-transfected into 293T cells using Lipofectamine TM 2000. Seventy-two hour later, the supernatant of the 293T cells was collected and purified by ultracentrifugation to obtain lentiviral particles carrying Serpin E1. Finally, viral titers were determined using a Luminescent assay.

Subsequently, CAFs and Hs738 cells (2 × 10^4^) were infected with Serpin E1-EGFP lentivirus and an EGFP-expressing control lentivirus. After 12 h, the culture medium was replaced. Once an infection efficiency of over 90% was reached through microscopic observation of EGFP fluorescence, puromycin (3 μg/ml) was introduced for selection in the cultures. Following a two-week period, stable cell lines overexpressing Serpin E1 were established for subsequent detection of Serpin E1 expression.

### Western blotting

The western blotting procedure was conducted according to standard protocols. Cells were lysed for 10 min with RIPA lysis solution (89,901, ThermoFisher, USA) supplemented with 1% protease inhibitors (#11873580001, Roche, USA). Subsequently, the lysates were subjected to electrophoresis in 12% SDS-PAGE gels after boiling at 100 °C for 10 min. The proteins were transferred to a PVDF membrane (Millipore, USA). The membranes were then pretreated with nonfat milk and subsequently incubated with primary and secondary antibodies. Protein bands were detected using ECL luminescent solution (Millipore), followed by quantification using Image J software. The detailed information regarding the antibodies utilized can be found in Additional file 1: Table S1.

### Immunohistochemistry (IHC)

Subcutaneous xenograft tumors and intraperitoneal tumors were excised from nude mice that were killed by an overdose of anesthesia. The tumors were fixed in 10% formalin, followed by paraffin embedding and sectioning into 5-μm thick slices. Deparaffinization of the slices was performed using xylene, followed by rehydration with a series of increasing ethanol concentrations. Antigen retrieval was achieved through high-pressure treatment in 0.01 M citrate buffer (pH 6.0), while endogenous peroxidases were inhibited using 3% H_2_O_2_. After that, the slices were incubated at 4 ℃ overnight with primary antibodies, including Ki67, Serpin E1, CD31, and VEGFA, followed by a 2-h incubation with HRP-conjugated secondary antibodies at room temperature. The signals were then visualized with a DAB kit (ab64238, Abcam, UK), The DAB-visualized slices were counterstained in hematoxylin, acid alcohol, and deionized water in sequential order. Subsequently, the slices underwent dehydrated in graded ethanol and cleared with xylene. Microscopic image was captured. Staining was quantified with Image J analysis software, and IHC score was determined by multiplying the percentage score with the intensity score. Details of antibodies used for immunohistochemistry are provided in Additional file 1: Table S1.

### Immunofluorescence

Cells, including *H. pylori*-infected CAFs, HUVECs co-cultured indirectly with Hs738 overexpressing Serpin E1, and recSerpin E1-treated HUVECs, were grown in 24-well plates on a cover slide for 72 h and fixed using 4% paraformaldehyde. Following permeabilization with 0.3% Triton X-100 and blocking with 5% BSA (#15260037, ThermoFisher), anti-Serpin E1 and anti-VEGFA antibodies were added to the slide overnight at 4 °C. This cover slide was then treated using a secondary antibody conjugated to fluorochrome for 2 h, and cell nucleus was stained by DAPI. Following images were taken, the integrated immunofluorescence (IF) intensity was quantified using Image J software.

For polychromatic immunofluorescent staining, tumor tissues from nude mice were fixed in 4% paraformaldehyde, embedded in paraffin, and sectioned into slices with a thickness of 5 cm. Then, the slices were stained with a four-color multiplex fluorescence immunohistochemical staining kit (abs50012, Absin) following the manufacturer's instructions. After dewaxing, clearing, and rehydrating steps, microwave-assisted antigen retrieval was performed using a sodium citrate buffer, followed by blocking of endogenous peroxidase activity with 0.3% H_2_O_2_ and washing with TBST containing 5% goat serum. Next, the slices were incubated with primary antibodies and subsequently treated with HRP-labeled secondary antibodies (goat anti-rabbit/mouse). The slices were developed using the fluorescent dye provided in the kit. Subsequently, the slices underwent another round of antigen retrieval step followed by incubation with another primary antibody until complete antigen staining was achieved. Finally, nuclear visualization was achieved by counterstaining the slices with DAPI. Confocal images were acquired using a confocal microscope, and relative fluorescence intensity was quantified utilizing Image J software. The details of antibodies used can be found in Additional file 1: Table S1.

### Human cytokine array

After infecting CAFs with *H. pylori* for 6 h at an MOI of 50, the free and dead *H. pylori* were thoroughly washed with PBS. The cultures were continued for another 7 days. The supernatants from CAFs with or without *H. pylori* infection were collected to determine cytokine levels using a Proteome Profiler Human Cytokine Array Kit (ARY005B, R&D Systems, USA), comprising 36 cytokines, following the manufacturer’s protocol. Briefly, the arrays spotted on nitrocellulose membranes were blocked at room temperature for 1 h with blocking buffer. Concurrently, the cell supernatants and a cocktail of biotinylated detection antibody were mixed and incubated for 1 h at room temperature, followed by overnight incubation at 4 °C with the membrane. Subsequently, the membranes were incubated with HRP-conjugated streptavidin at room temperature for 30 min, followed by exposure to ECL substrate (Promega, USA). Blots were visualized by chemiluminescence utilizing ECL. A comprehensive list of human cytokines included in this array refer to our previous publication [16].

### Cell Counting Kit-8 (CCK-8) assay

GC cells (0.5 × 10^4^) treated with recSerpin E1 (1 and 10 ng/ml) were grown in 96-well plates. After culturing for different time points, 10μl CCK-8 (CK04, Dojindo, Japan) were added to each well. The absorbance at 450 nm was measured after a two-hours incubation at 37 °C.

### Colony formation assay

CAFs (4.5 × 10^3^) were mixed with AGS cells (5 × 10^2^) at a 9:1 ratio and seeded in 12-well plates. Alternatively, GC cells treated with recSerpin E1 were plated in 6-well plates. Following a 15-day incubation at 37 °C, cell colonies were stained with 0.5% crystal violet solution and counted. In the co-culture system, only cancer cells have the capacity to form colonies, whereas CAFs exhibited loose growth. Consequently, the colony number represents the proliferative ability of cancer cells.

### Transwell assay

In 24-well plates, Serpin E1 knockdown CAFs (1 × 10^5^), Serpin E1 overexpression CAFs (1 × 10^4^), and recSerpin E1 (1ng/ml) were added to the lower chamber of Transwell inserts. GC cells (0.5–1 × 10^4^) were seeded to the upper chamber with or without Matrigel (#356234, Corning, NY, USA). After 48 h of co-culture for invasion assays and 24 h for migration assays or treatment with recSerpin E1 for 72 h, the migrated and invaded cells on the lower surface of the membrane were fixed by 4% paraformaldehyde solution, stained by 0.1% crystal violet, and counted from five randomly selected fields.

### In vivo tumorigenesis assay

The animal research protocol was approved by Guizhou Medical University's animal ethics committee (No: 1702155). Primary GC cells (1 × 10^6^) and CAFs or Hs738 cells (4 × 10^6^) stably expressing Serpin E1-EGFP (non-fusion) or EGFP (Control) were mixed at a ratio of 1:4 in 250 μl of PBS and subsequently subcutaneously and peritoneally injected into male nude mice (4-week-old, *n* = 3 or 5 per group) obtained from Chongqing Tengxin Biotechnology (China). Subcutaneous tumor growth was assessed by monitoring tumor volume (V = 1/2 × length × width^2^) every two days. On the 24th or 28th day after cell injection, all mice were killed with an excessive dosage of anesthesia using 1% pentobarbital sodium at a dosage of 100 mg/kg (intraperitoneal injection), and both subcutaneous tumors and peritoneal tumor nodules were collected for HE, IHC, and immunofluorescence staining.

### Chemotaxis assay

In a 6-well plate, CAFs or Hs738 cells were incubated with *H. pylori* for 6 h at 50 MOI. Subsequently, the medium was exchanged for fresh medium to eliminate any free or dead bacteria. Matrigel was diluted in a ratio of 1:8 with DMEM and loaded onto an 8 μm pore size membrane in the upper chamber of Transwell inserts, which were placed within a 24-well plate. Following Matrigel polymerization, HUVECs (1 × 10^4^) were seeded on top of the Matrigel, and fibroblasts, either infected or uninfected with *H. pylori*, their respective conditioned medium (diluted by half using fresh medium), and recSerpin E1 (1 ng/ml) were added into the lower chambers. After incubation for 48–72 h, the Transwell inserts were removed and fixed. The migrated HUVECs were stained with 0.1% crystal violet and counted.

### Tube formation assay

Matrigel was diluted 1:4 with DMEM and incubated at 37 °C for 2 h in a 12-well plate or the Transwell lower chamber within a 24-well plate. After polymerization of the Matrigel, HUVECs suspended in DMEM (10% FBS) were seeded on top of the Matrigel in each plate. Subsequently, recSerpin E1 (1 ng/ml) and an anti-Serpin E1 antibody (2 μg/ml) were added into each well with a time interval of 6 h in the 12-well plate. Alternatively, Serpin E1-overexpressed CAFs (1 × 10^5^) were grown in the upper chamber with a membrane of 0.4-μm pore size (preventing cellular migration through the membrane) in a 24-well plate. After incubation for 24 or 72 h, the formation of tubular structures by HUVECs in tissue culture plates or the lower Transwell chambers was observed and documented using an inverted microscope. The mean length and branch point of tubes from three independent wells were quantified using Image J software.

### Enzyme-linked immunosorbent assay (ELISA)

CAFs were grown in 6-well plates and infected with *H. pylori* for 6 h. Free-floating bacteria were eliminated by washing with PBS. After 3 days, the culture supernatant was collected to determine Serpin E1 levels using a human Serpin E1 ELISA kit (EK1136, MultiSciences, China). Alternately, HUVECs were co-cultured with Serpin E1-overexpressed or control Hs738 cells in both direct and indirect co-culture systems, where CAFs were grown in the upper chambers of Transwell insert and HUVECs in the lower chambers. After 48 h, the culture supernatants were collected to determine VEGFA levels using a human VEGFA ELISA kits (EK183, MultiSciences).

### Statistical analysis

The analyses of the data were performed by the SPSS 16.0 software. Image J software was employed to analyze the images from tube formation and IHC experiments. GraphPad Prism 5 was used for graph generation. An unpaired two-tailed Student’s t-test was utilized to compare the two groups, while two-way and one-way analysis of variance were employed to compare multiple groups. Each experiment underwent a minimum of three repetitions. All images shown were representative images from three independent tests or samples. Data is represented as mean ± standard deviation, with statistically significant defined as *p* < 0.05.

## Results

### Infection with *H. pylori* upregulates the expression of Serpin E1 in CAFs to promote GC cell proliferation, migration, and invasion

**Fig. 1:**
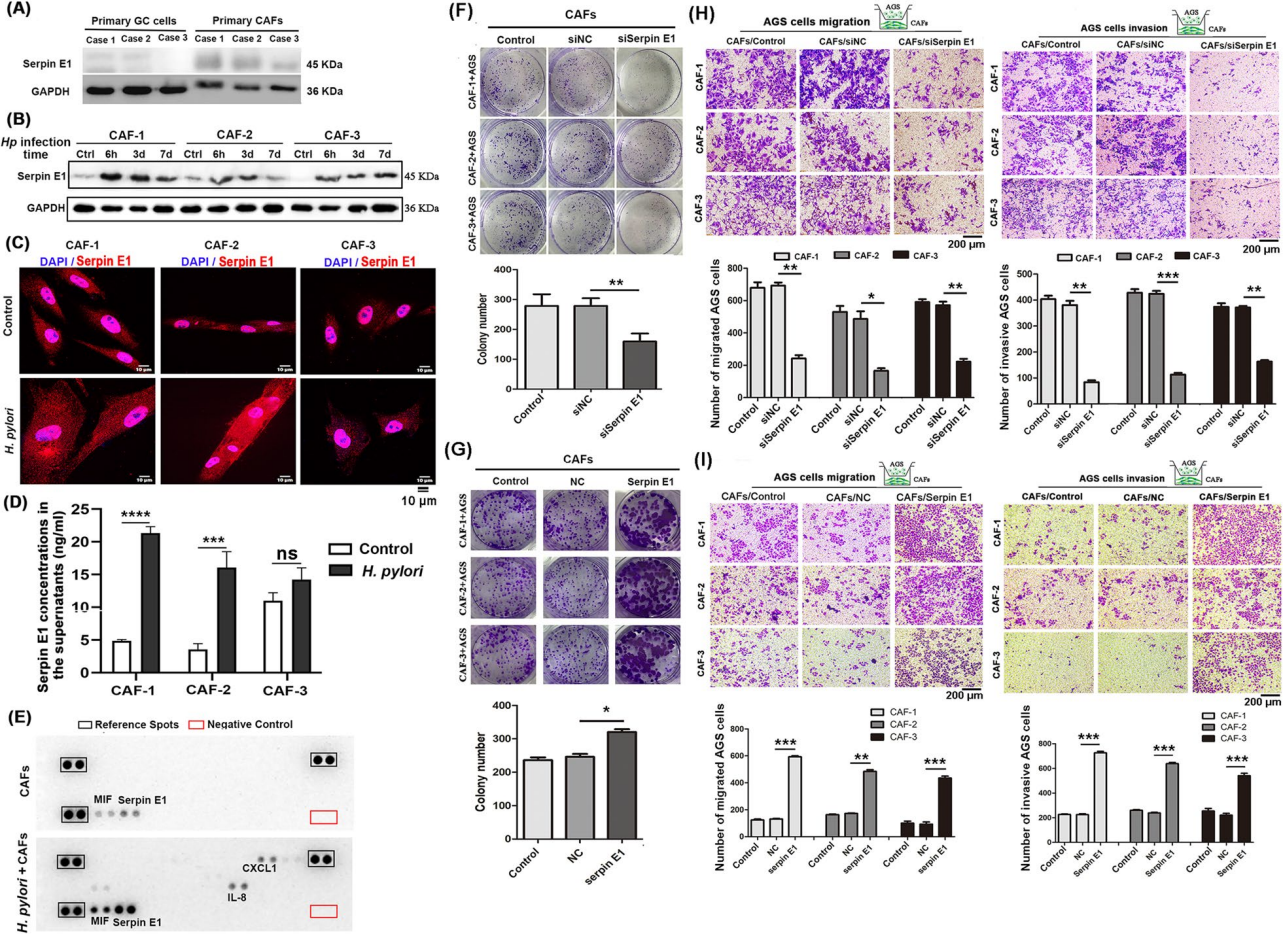
*H. pylori* upregulates Serpin E1 expression in CAFs to promotes the colony formation, migration, and invasion of AGS cells. **A** Expression of Serpin E1 was detected in primary GC cells and CAFs from the same patients by western blotting. **B**, **C** Expression of Serpin E1 was detected in *H. pylori*-infected CAFs at an MOI of 50 by western blotting (**B**) and immunofluorescence (**C**). Scale bar = 10 μm. **D** Serpin E1 concentrations in the supernatants were determined in CAFs infected with *H. pylori* for 3 days by ELISA. **E** Cytokines in the supernatants were measured using a human cytokine array after infecting CAFs with *H. pylori* for 7 days, following removal of free *H. pylori* at 6 h post-infection. MIF: macrophage migration inhibitory factor; CXCL1: C–X–C motif chemokine ligand 1; IL-8: interleukin-8. **F**, **G** Colony formation ability was evaluated in AGS cells co-cultured with Serpin E1 knockdown (**A**) and overexpression (**C**) CAFs. **H**, **I** The invasion and migration capacities were detected using the Transwell assay in AGS cells co-cultured with Serpin E1 knockdown (**B**) and overexpression (**D**) CAFs. Scale bar = 200 μm; *Hp*: *H. pylori*; Ctrl: Control; Line graphs and bar graphs show the mean ± SD; *p < 0.05, **p < 0.01, ***p < 0.001, and ****p < 0.001

In our previous studies, we successfully isolated three primary CAFs and GC cells from GC tissues of three patients, as well as a *H. pylori* GZ7 strain from a GC patient's stomach mucosa [16, 22]. We observed that Serpin E1 was predominantly expressed in CAFs but almost undetectable in GC cells derived from the same patients (Fig. 1A). Moreover, infection with *H. pylori* enhanced Serpin E1 expression in CAFs, particularly at 6 h and 3 days post-infection (Fig. 1B). The immunofluorescence analysis revealed a prominent increase of Serpin E1 in the cytoplasm of CAFs upon *H. pylori* infection, whereas it primarily localized to the nucleus under normal conditions (Fig. 1C). Additionally, the levels of secreted Serpin E1 in culture supernatants derived from *H. pylori*-infected CAFs were significantly higher compared to uninfected CAFs (Fig. 1D), indicating that infection with *H. pylori* promoted both expression and secretion of Serpin E1 by CAFs. Subsequently, a human cytokine array consisting of 36 cytokines was employed to determine the levels of cytokine in the culture supernatants of *H. pylori*-infected CAFs after 7 days, following removal of free *H. pylori* at 6 h post-infection. Among the panel of 36 cytokines examined, only Serpin E1, interleukin-8 (IL-8), C-X-C motif chemokine ligand 1 (CXCL1), and macrophage migration inhibitory factor (MIF) were upregulated in *H. pylori*-infected CAFs, with Serpin E1 exhibiting the highest increase (Fig. 1E).

CAFs have been confirmed to interact directly with cancer cells in the TME [23]. This encouraged us to test a functional role of Serpin E1 in mediating the crosstalk between CAFs and GC cells. Six CAFs with Serpin E1 knockdown and overexpression, along with their respective control cells, were generated (Additional file 1: Fig. S1) and co-cultured with AGS cells either directly or using a Transwell system, in which AGS cells were added into the upper chambers while CAFs were added into the lower chambers of Transwell inserts. Our results revealed that knockdown of Serpin E1 in CAFs significantly suppressed colony formation, invasion, and migration of AGS cells (Fig. 1F, H), whereas overexpression of Serpin E1 in CAFs produced opposite effects (Fig. 1G, I), suggesting that Serpin E1 derived from CAFs promoted a malignant phenotype of GC cell in vitro. In addition, Serpin E1 knockdown also inhibited CAFs growth and enhanced their apoptosis, while Serpin E1 overexpression exerted the converse effects (Additional file 1: Fig. S2).

### Recombinant human Serpin E1 (recSerpin E1) promotes GC cell growth, migration, and invasion

**Fig. 2:**
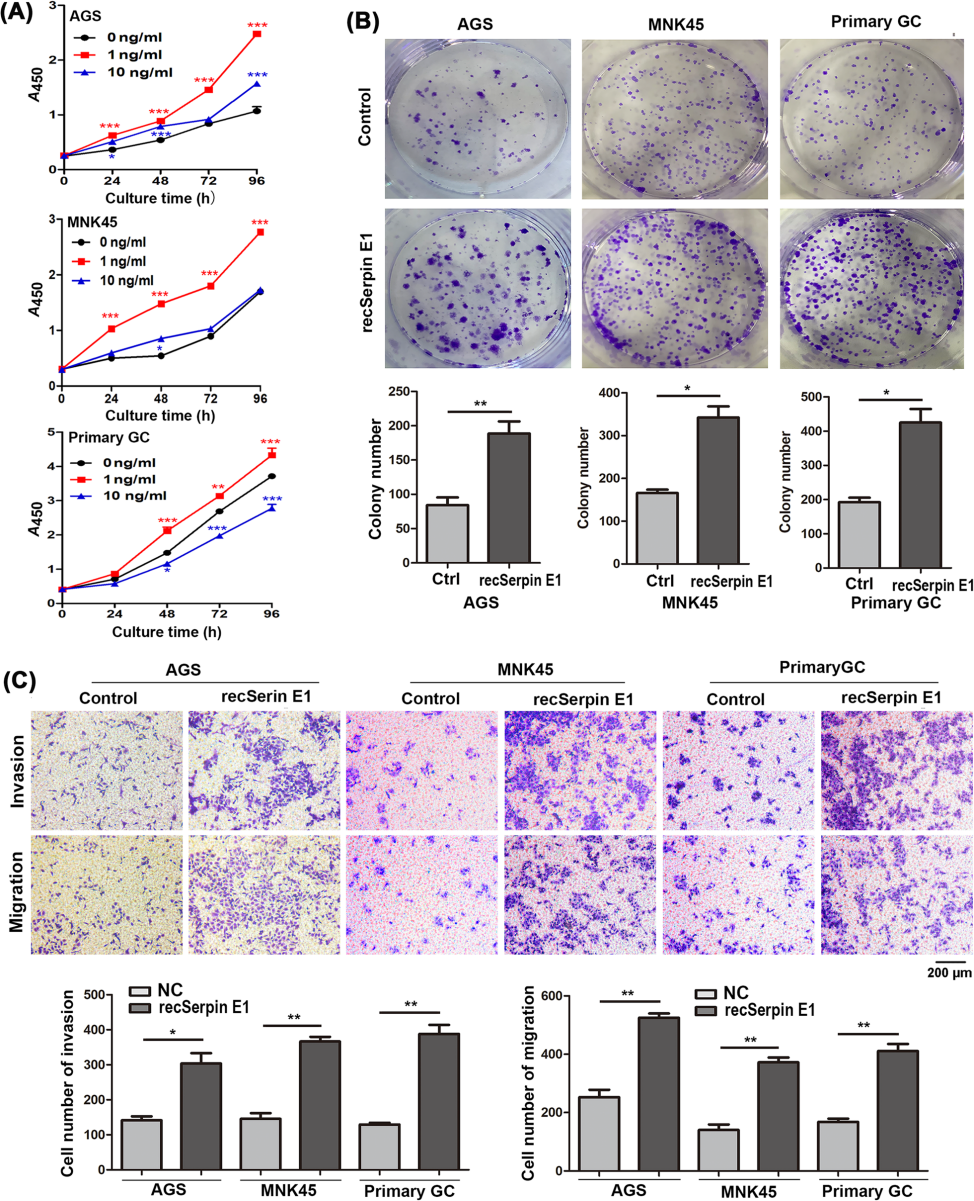
recSerpin E1 promotes the proliferation, migration, and invasion of GC cells. **A** recSerpin E1 promotes GC cell proliferation by CCK-8 assay. Line graphs represent the mean ± SD; *p < 0.05, **p < 0.01, and ***p < 0.001 vs. 0 ng/ml group. **B**, **C** recSerpin E1 (1 ng/ml) promotes colony formation (**B**) and migration and invasion (**C**) capacities of GC cells. Ctrl: Control; Scale bar = 200μm; Bar graphs show the mean ± SD; *p < 0.05, and **p < 0.01

To further confirm the tumor-promoting effects of CAFs-derived Serpin E1, AGS cells, MNK45 cells, and primary GC cells were exposed to recSerpin E1 at concentrations of 1 and 10 ng/ml. Consistent with the findings obtained from CAFs-derived Serpin E1, the exposure to 1 ng/ml recSerpin E1 significantly enhanced cell proliferation, colony formation, invasion, and migration in all three GC cell lines (Fig. 2A–C). However, treatment with 10 ng/ml recSerpin E1 did not induce an increase and even resulted in a decrease in GC cell growth (Fig. 2A). This observation suggests that high doses of recSerpin E1 may exert cytotoxicity on cultured cells.

### Fibroblast-derived Serpin E1 promotes xenograft tumor growth of primary GC cells via inducing angiogenesis in nude mice

**Fig. 3:**
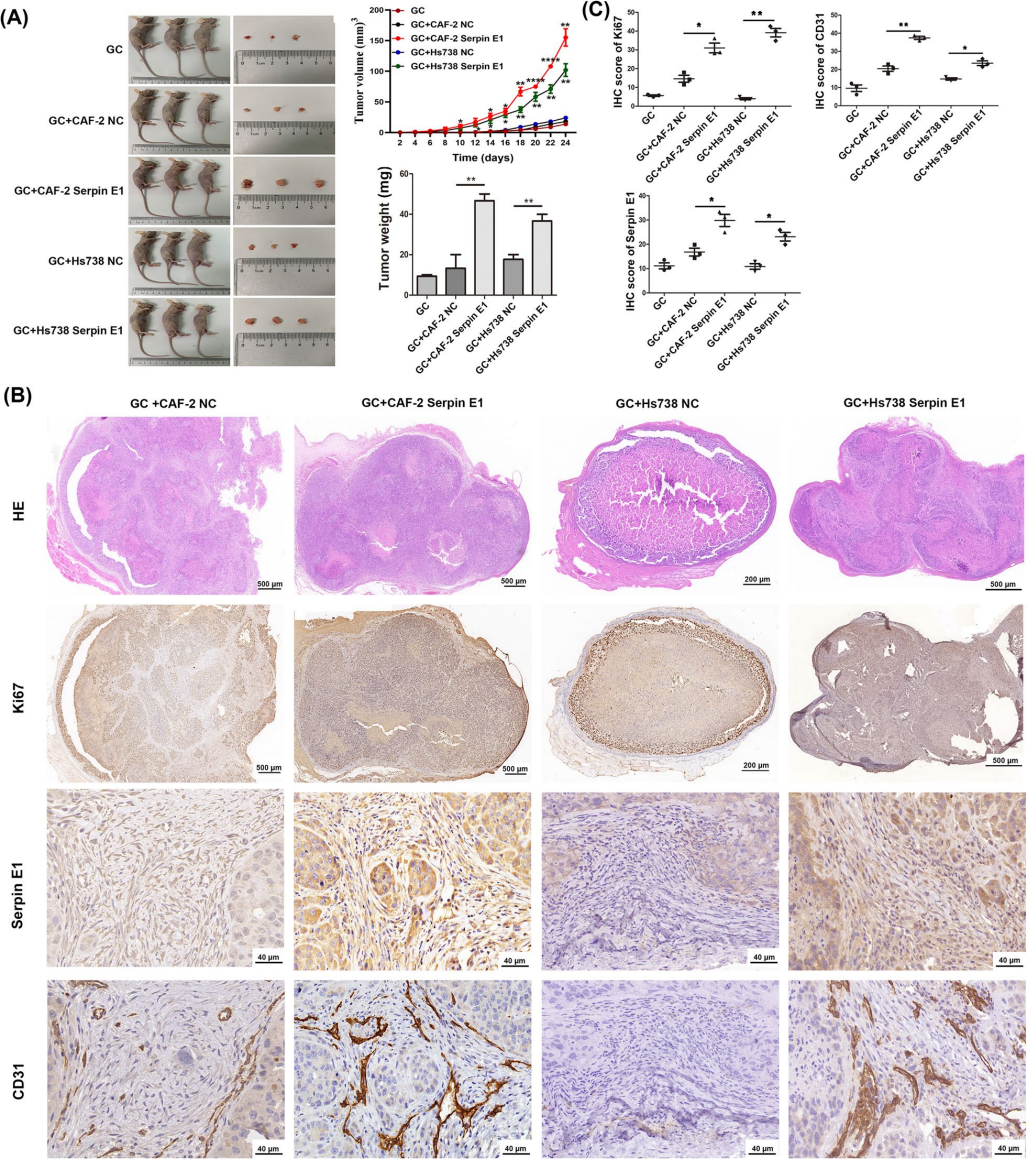
Co-inoculation of Serpin E1-overexpressed fibroblasts and primary GC cells promotes subcutaneous tumor growth by inducing angiogenesis in nude mice. **A** Tumorigenicity assays show that fibroblast-derived Serpin E1 enhances the tumorigenic potential of primary GC cells. Mice were killed 24 days after co-inoculation. Tumor volume was calculated using the formula: 1/2 × length × width^2^. Line graphs represent the mean ± SD (n = 3); *P < 005, **P < 0.01, and ***P < 0.001 vs. GC group. **B** HE staining and IHC staining for Ki67, Serpin E1, and CD31 on sections from the subcutaneous tumor (n = 3). Scale bars: 500 μm or 200 μm (upper 2 rows) and 20 μm (lower 2 rows). **C** Quantification of IHC staining using Image J software. Bar graphs and dot plots show the mean ± SD (n = 3); *p < 0.05, and **p < 0.05

After confirming the pro-tumorigenic role of both fibroblast-derived Serpin E1 and recSerpin E1 in vitro, we further investigated their function in driving tumor growth and progression in vivo. Fibroblasts overexpressing Serpin E1, including CAFs and Hs738 cells, or fibroblasts expressing EGFP (NC) were mixed with primary GC cells at a 4:1 ratio (fibroblasts: GC cells) and co-implanted subcutaneously into nude mice. Although xenograft tumor volume and weight were higher in the GC + fibroblast NC groups compared to the only GC groups, no statistically significant differences were observed between these two groups. However, overexpression of Serpin E1 in fibroblasts significantly enhanced subcutaneous tumor growth when compared to the GC + fibroblast NC groups, as evidenced by significantly increased tumor volume and weight in the GC + fibroblast Serpin E1 groups (Fig. 3A, Additional file 1: Fig. S3). Histological examination using HE staining and IHC staining of Ki67 revealed focal necrosis within the central region of xenograft tumors in the GC + fibroblast NC groups; however, overexpression of Serpin E1 in fibroblasts alleviated central tumor necrosis (Fig. 3B, C).

Angiogenesis is crucial for tumorigenesis and tumor progression by supplying oxygen and nutrients to growing tumors. CD31, expressed in vascular endothelial cells, is an essential marker for this process [24]. We observed elevated levels of CD31 expression within the vascular endothelial cells of tumor tissues from the GC + fibroblast Serpin E1 groups compared to those from the GC + fibroblast NC groups (Fig. 3B, C), indicating that fibroblast-derived Serpin E1 induces angiogenesis. In addition, we also noted a significant increase in Serpin E1 expression within cancer cells, especially in the GC + fibroblast Serpin E1 groups (Fig. 3B, C), despite the absence of Serpin E1 expression in cancer cells cultured in vitro.

### Fibroblast-derived Serpin E1 promotes peritoneal dissemination of primary GC cells via enhancing VEGFA expression and angiogenesis in nude mice

**Fig. 4:**
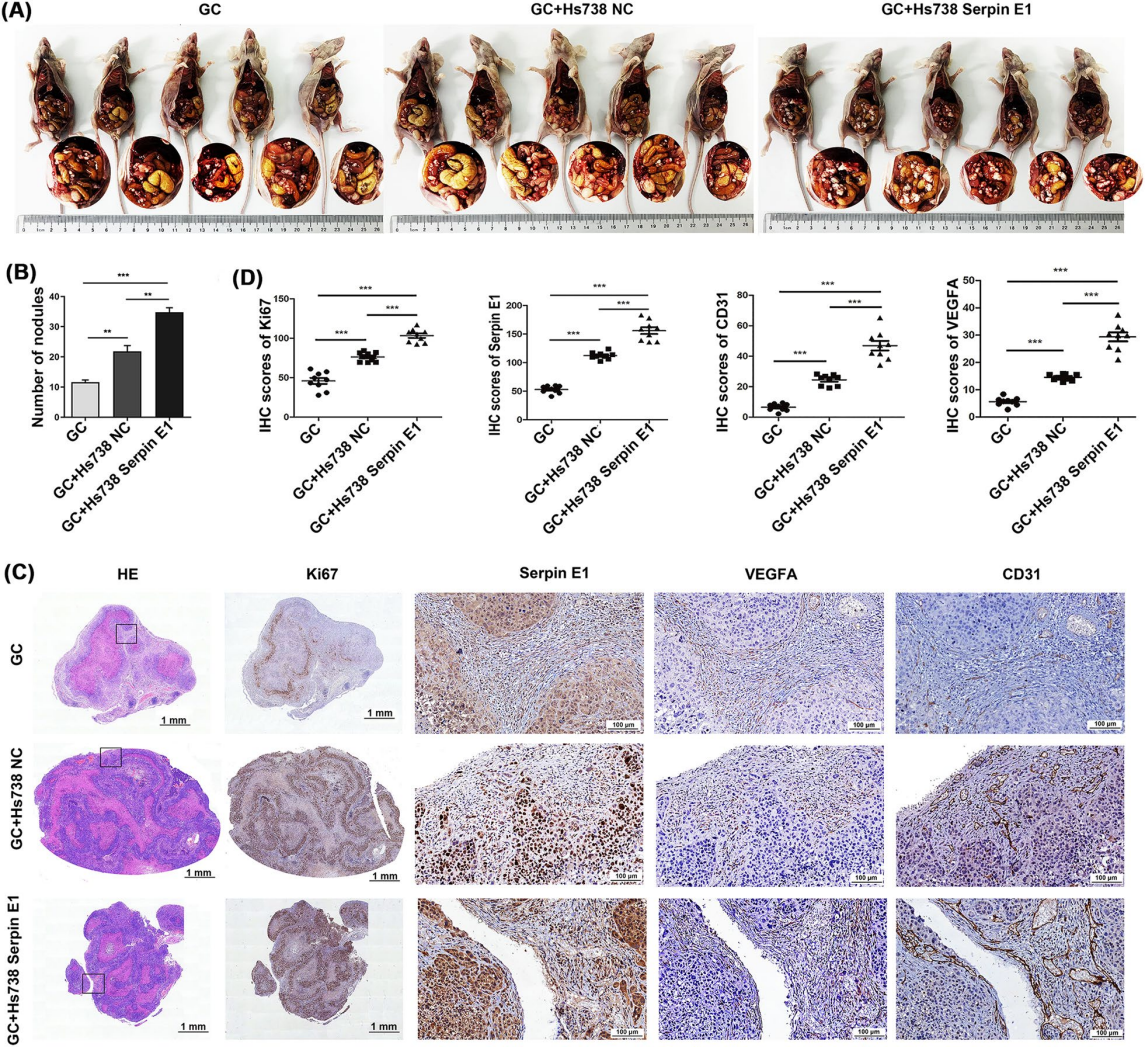
Co-injection of Hs738 overexpressing Serpin E1 and primary GC cells promotes peritoneal dissemination of cancer cells by promoting VEGFA expression and angiogenesis in nude mice. **A** Hs738 cells-derived Serpin E1 facilitates peritoneal dissemination of primary GC cells. Mice were killed 28 days after injection. **B** Quantitative analysis of tumor nodules in the peritoneal cavity by counting. **C** HE staining and IHC analysis of Ki67, Serpin E1, VEGFA, and CD31 on sections from the peritoneal tumor. Scale bars: 1 mm (left 2 columns) and 100 μm (right 3 columns). **D** Quantification of IHC staining using the Image J software (n = 9 tumor nodules per mouse). Bar graphs and dot plots show the mean ± SD, n = 5; **p < 0.05, and ***p < 0.001

Peritoneal dissemination is an excellent indicator of GC progression and metastasis. Therefore, Fibroblast (Hs738 cells) overexpressing Serpin E1 (Hs738 Serpin E1) or the negative control (Hs738 NC) were intraperitoneally co-injected with primary GC cells into nude mice at a 4:1 cell ratio. Our findings revealed an increased tumor nodules within the peritoneal cavity of mice co-injected with Hs738 Serpin E1 and GC cells, compared to those co-injected with Hs738 NC and GC cells and injected with GC cells alone (Fig. 4A, B). Furthermore, we observed a higher number of tumor nodules in the GC + Hs738 NC group compared to the GC group (Fig. 4A, B), suggesting that fibroblasts could enhance peritoneal spreading of GC cells, with Serpin E1 overexpression in fibroblasts exhibiting the most potent promotion effects. Similar to subcutaneous xenograft tumors, all three groups displayed focal necrosis within tumor nodules; however, co-injection of GC cells and Hs738 cells could alleviate tumor necrosis, particularly in the GC + Hs738 Serpin E1 group (Fig. 4C). The positive staining of Ki67 within these nodules further supported this finding (Fig. 4C, D). Moreover, as the expression of Serpin E1 in fibroblasts increased progressively from the GC group to the GC + Hs738 NC group and finally to the GC + Hs738 Serpin E1 group, the expression of angiogenesis-related factors VEGFA and CD31 also exhibited progressive upregulation (Fig. 4C, D), indicating that fibroblast-derived Serpin E1 promote neovascularization within tumors. Additionally, all three groups showed the expression of Serpin E1 by GC cells within tumor nodules; however, the highest expression levels were observed in the GC + Hs738 Serpin E1 group (Fig. 4C, D).

### Fibroblast-derived Serpin E1 promotes Serpin E1 expression in GC cells in xenograft tumors of nude mice

**Fig. 5:**
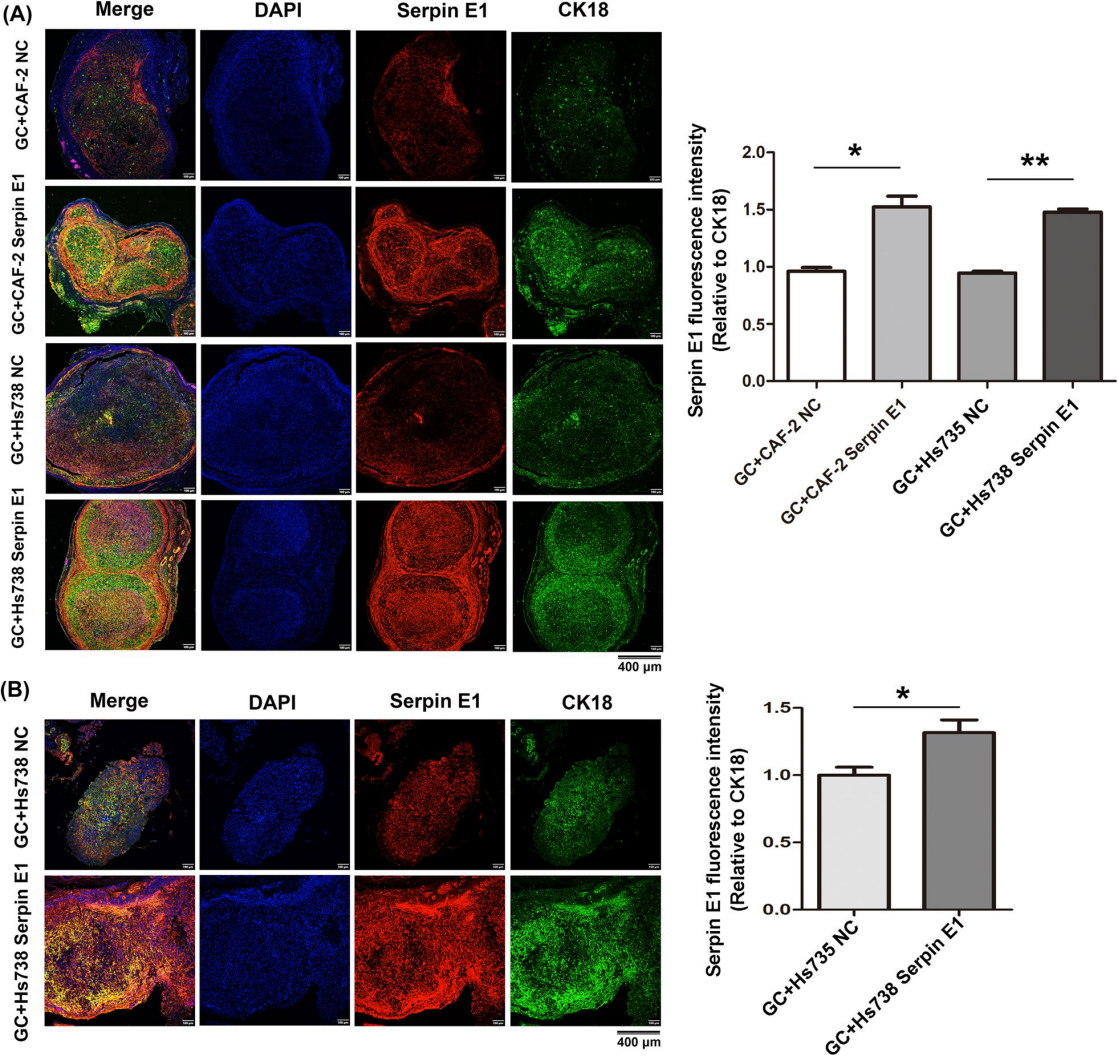
Fibroblast-derived Serpin E1 induces Serpin E1 expression in cancer cells within subcutaneous and peritoneal tumors of nude mice. **A**, **B** Expression of Serpin E1 and CD18 in subcutaneous (**A**) and peritoneal (**B**) tumors was assessed through polychromatic immunofluorescent staining using confocal microscopy. The fluorescence intensity was quantified using Image J software. The ratio of red fluorescence (Serpin E1) to green fluorescence (CK18) indicates Serpin E1 expression in cancer cells. DAPI (blue fluorescence) was used for labeling the cell nucleus. Scale bars: 400 μm. Bar graphs show the mean ± SD (n = 3); *p < 0.05, and **p < 0.01

In our previous study, we confirmed in vitro that CAFs induced Serpin E1 expression in cancer cells through direct contact and indirect co-culture within a Transwell system using IHC and western blotting [16]. In the present study, we also observed the strongest straining for Serpin E1 in cancer cells of subcutaneous and peritoneal tumor when co-injected with fibroblasts overexpressing Serpin E1 in nude mice. To further validate this observation, polychromatic immunofluorescent staining was employed to assess Serpin E1 expression in cancer cells labeled with cytokeratin 18 (CD18), an epithelial cell-specific marker, within tumors. The results revealed a significant increase in merged fluorescence intensity of Serpin E1 and CK18 in both subcutaneous and peritoneal tumors co-injected with GC cells and Serpin E1-overexpressed fibroblasts compared to those co-injected with GC cells and control fibroblasts (Fig. 5A, B). The quantitative analysis showed that the fluorescence intensity of Serpin E1, relative to CK18, was significantly higher in the co-injection groups of GC cells and Serpin E1-overexpressed fibroblasts compared to the co-injection groups of GC cells and control fibroblasts in both subcutaneous and peritoneal tumors (Fig. 5A, B), while no significant difference was observed in DAPI fluorescence intensity between the two corresponding compared groups (Additional file 1: Fig. S4). These findings further confirmed our hypothesis that fibroblast-derived Serpin E1 induced its expression in cancer cells.

### Fibroblast-derived Serpin E1 increases migration, proliferation, tube formation, and VEGFA expression of HUVECs

**Fig. 6:**
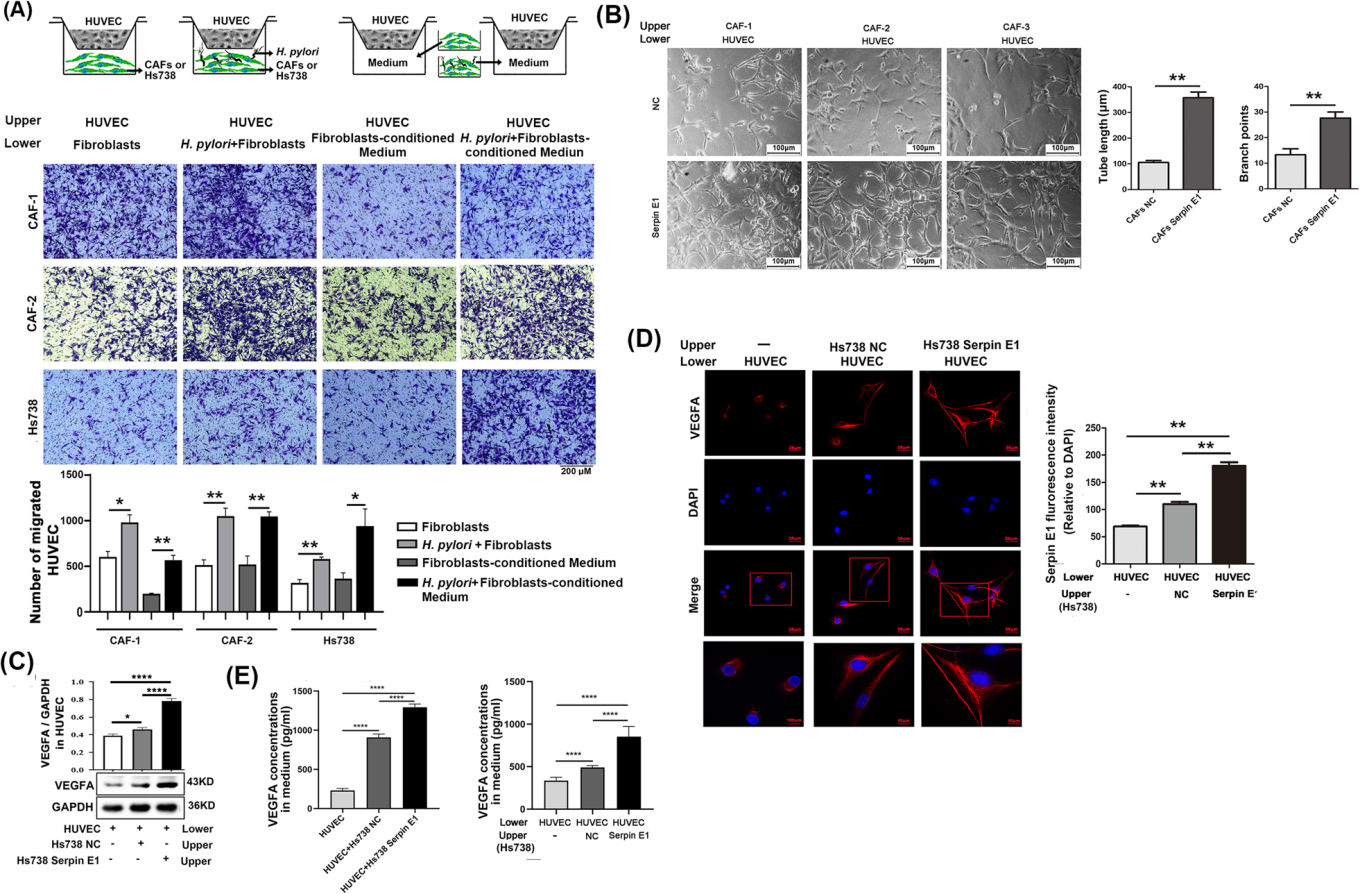
Infection with *H. pylori* and Serpin E1 overexpression in fibroblasts promote the migration, tube formation, and VEGFA expression of HUVECs. **A** Chemotactic migration of HUVECs was assessed by Matrigel-coated Transwell assay, wherein HUVECs were seeded in the upper chamber while fibroblasts with and without *H. pylori* infection or their conditioned medium were added into the lower chamber. Scale bar = 200 μm. **B** Tube formation of HUVECs on Matrigel was assessed by a Transwell co-cultured model, where CAFs overexpressing Serpin E1 were added in the upper chamber and HUVECs were seeded in the Matrigel-covered lower chamber. Scale bar = 100 μm. **C** Western blotting analysis of VEGFA in HUVECs co-cultured with Hs738 cells overexpressing Serpin E1, directly and using a Transwell system. **D** Immunofluorescence staining of VEGFA in HUVECs co-cultured with Serpin E1-overexpressed Hs738 cells using a Transwell insert. Scale bars: 25 μm and 10 μm (bottom row). **E** VEGFA levels were determined in the culture supernatants of HUVECs directly and Transwell co-cultured with Serpin E1-overexpressed Hs738 cells by ELISA. Bar graphs show the mean ± SD (n = 3); *p < 0.05, **p < 0.01, ***p < 0.001, and ****p < 0.001

Tumor neovascularization involves the proliferation, migration, and tube formation of endothelial cell [25]. Therefore, *H. pylori*-infected fibroblasts (including CAFs and Hs738 cells) and their conditioned media were co-incubated with HUVECs using Transwell inserts, where HUVECs were seeded to the Matrigel-coated upper chamber while fibroblasts and conditioned media were added to the lower chamber. Both infection of fibroblasts with *H. pylori* and the conditioned medium significantly increased the migration of HUVECs from the Transwell upper to lower chambers (Fig. 6A). Furthermore, Serpin E1 derived from fibroblasts not only stimulated tube formation of HUVEC by increasing total tube length and branch point numbers but also promoted HUVEC proliferation by facilitating cell cycle progression (Fig. 6B, Additional file 1: Fig. S5).

VEGFA is a potent pro-angiogenic growth factor involved in cancer initiation and development [24]. We found that overexpression of Serpin E1 in Hs738 cells could promote VEGFA expression in co-cultured HUVECs within a Transwell system (Fig. 6C). This finding was further confirmed by immunofluorescence staining for VEGFA in co-cultured HUVECs; here we found a significant increase in size and fluorescence intensity of VEGFA-positive cells when co-cultured with Hs738 Serpin E1 (Serpin E1 overexpression) compared to co-culture with Hs738 NC or solely culture of HUVECs (Fig. 6D). Similarly, there was a significant enhancement in the levels of secreted VEGFA in the supernatants from direct or indirect co-culture of HUVECs and Hs738 cells overexpressing Serpin E1 (Fig. 6E). Moreover, both expression and secretion of VEGFA were elevated when comparing co-cultured group of HUVECs and Hs738 cells with solely cultured group of HUVECs (Fig. 6C–E). Additionally, the knockdown of Serpin E1 decreased VEGFA expression while its overexpression increased VEGFA expression in CAFs (Additional file 1: Fig. S5). These findings suggested that HUVECs and CAFs may be crucial sources of VEGFA within the TME of GC.

### recSerpin E1 enhances the migration, tube formation, and p38 MAPK-dependent VEGFA expression in HUVECs

**Fig. 7:**
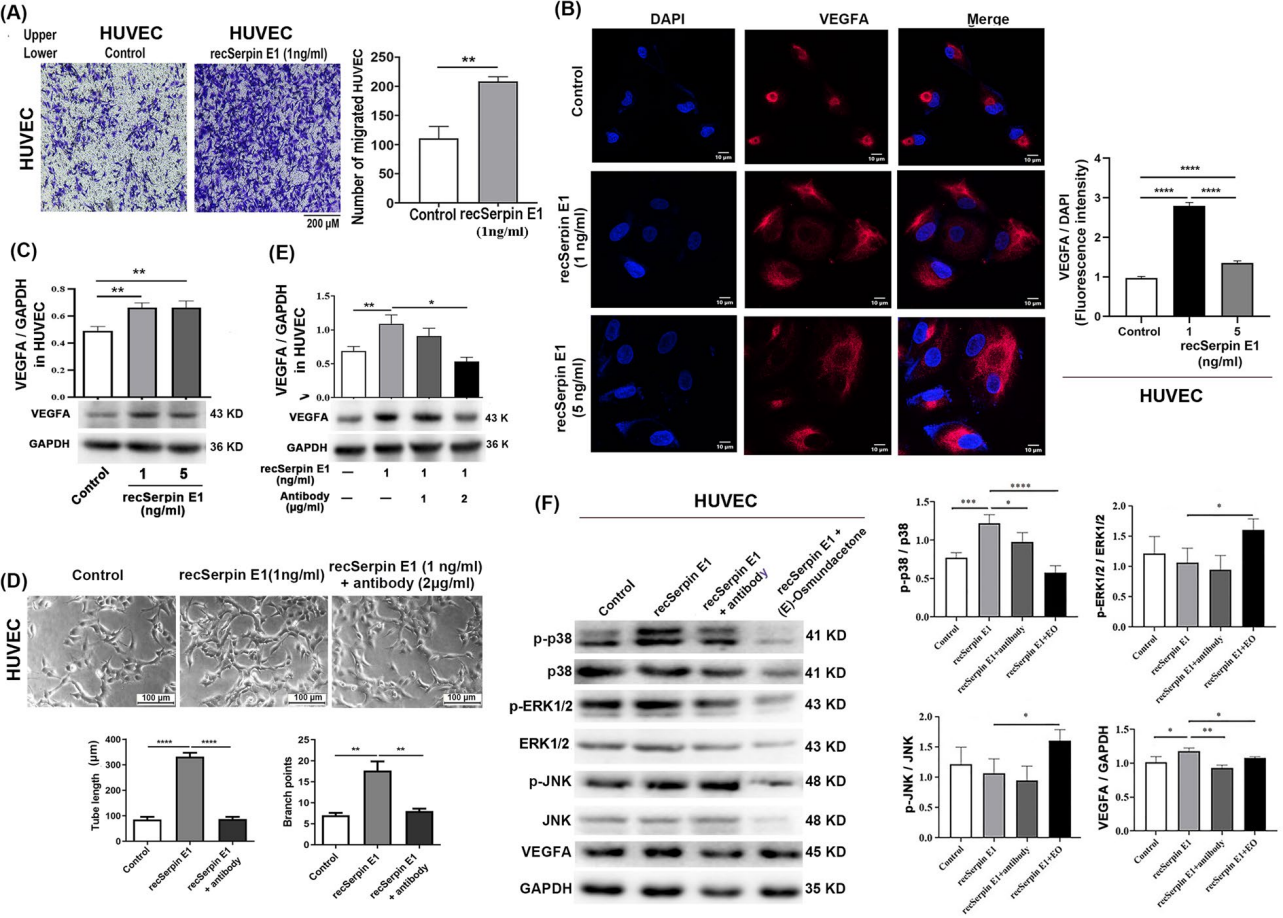
recSerpin E1 promotes the migration, tube formation, and p38 MAPK-dependent VEGFA expression in HUVECs. **A** recSerpin E promotes chemotactic migration of HUVECs by Matrigel-coated Transwell system. **B**, **C** VEGFA expression was determined in HUVECs treated with recSerpin El by immunofluorescence staining (**B**) and Western blotting (**C**). Scale bar = 10μm. **D**, **E** Tube formation on Matrigel (**D**) and VEGFA expression (**E**) were assessed in HUVECs co-treated with recSerpin E1 and anti-Serpin E1 antibody at a 6-h interval. Scale bar = 100μm. **F** Expression of p-p38, p-ERK1/2, p-JNK, and VEGFA was detected by Western blotting in HUVECs co-treated with recSerpin E1 (1 ng/ml) and anti-Serpin E1 antibody (2 μg/ml) or MAPK phosphorylation inhibitor (E)-osmundacetone (2 μM) at a 24-h interval. Bar graphs show the mean ± SD; *p < 0.05, **p < 0.01, ***p < 0.001, and ****p < 0.0001

To further elucidate the underlying mechanism behind Serpin E1-mediated promotion of GC, recSerpin E1 was used to treat HUVECs at a concentrate of 1 and/or 5 ng/ml. The results demonstrated that recSerpin E1 significantly increased HUVEC migration from the Transwell upper chamber through a Matrigel-coated membrane to the lower chamber (Fig. 7A). Immunofluorescence staining and western blotting revealed that treatment with recSerpin E1 enhanced VEGFA expression in HUVECs (Fig. 7B, C). Moreover, recSerpin E1 induced a greater tube formation of HUVECs, as evidenced by a significant increase in both total tube length and branch point numbers compared to the control group (Fig. 7D). Furthermore, the addition of anti-Serpin E1 antibody (2 μg/ml) suppressed Serpin E1-mediated VEGFA expression and tube formation in HUVECs (Fig. 7D, E).

VEGFA activation and subsequent tumor metastasis are tightly linked to three key MAPK signaling pathways: p38, ERK1/2, and JNK [26]. We found that recSerpin E1 enhanced the phosphorylation of p-38 MAPK and the expression of VEGFA but had no significant effects on ERK1/2 and JNK MAPKs compared to the controls in HUVECs. The addition of anti-Serpin E1 antibody (2 μg/ml) or (E)-osmundacetone, a universal inhibitor of MAPK phosphorylation, effectively suppressed recSerpin E1-induced p-38 phosphorylation and VEGFA expression (Fig. 7F), indicating that Serpin E1 promoted VEGFA activation via the p38 MAPK pathway in HUVECs. In addition, treatment with (E)-osmundacetone elevated the phosphorylation of ERK1/2 and JNK with unknown reason.

## Discussion

The infection with *H. pylori* stimulates the production and secretion of chemokines and cytokines by epithelial cells into the ECM, thereby contributing to tumor initiation and progression. However, Serpin E1 has received limited attention in this context. Microarray and RNA sequencing data analysis from GC tissues revealed that Serpin E1 was overexpressed in cancer tissues compared to peri-cancer tissues, and its overexpression was related to poorer patient survival [27]. Suh et al. [28] reported that Serpin E1 was upregulated over twofold in GC patients with advanced stages and lymph node metastases. Previous studies primarily focused on Serpin E1 expression in cancer cells [29]. However, the present study demonstrates that Serpin E1 expression is predominantly found in CAFs rather than cancer cells in GC tissues from the same patients, and *H. pylori* infection specifically promotes the expression of Serpin E1 in CAFs.

The expression of Serpin E1 in *H. pylori*-infected CAFs was found to be independent of other chemokines and cytokines, despite the known regulation of Serpin E1 transcription by transforming growth factor-beta 1 (TGF-b1), interleukin (IL)-6, and tumor necrosis factor (TNF)-α, in renal and adipose tissues [30, 31]. Our study, using a human cytokine array consisting of 36 chemokines and cytokines such as IL-6, TNF, IL-8, and others, revealed that only Serpin E1, MIF, IL-8, and CXCL1 were detected in the supernatant of *H. pylori*-infected CAFs. Notably, among these factors examined, Serpin E1 exhibited the highest abundance, suggesting a directly stimulation of its production by CAFs upon *H. pylori* infection. This finding is partially supported by Keates et al. [32], who reported that soluble factors from media separated from bacteria using a 0.1-μm filter did not affect the release of Serpin E1 by AGS cells.

Our study, including previous research [16], confirms that the interaction between fibroblasts and GC cells induces the expression of Serpin E1 in cancer cells. Importantly, the overexpression of Serpin E1 in fibroblasts has the strongest induction effects despite its low expression in GC cells in vitro. Furthermore, the overexpression of Serpin E1 in both fibroblast and GC cell enhance cancer cell proliferation, invasion, and migration in vitro, as well as subcutaneous tumor growth and/or intraperitoneal dissemination in nude mice. Conversely, knocking down Serpin E1 in fibroblasts exhibits the opposite effect in vitro. Similar results are also obtained when treating GC cells with recSerpin E1. Collectively, our findings reveal that *H. pylori* infection promotes Serpin E1 expression in fibroblasts, which then interact with GC cells to induce its expression in cancer cells, thereby jointly contributing to GC development and progression. However, the underlying mechanism remains unclear.

Serpin E1 has three distinct conformational states that are determined by the state of its reactive center loop (RCL) state: a metastable active form with an intact RCL exposed at the molecule surface, capable of binding to and inhibiting uPA/tPA activity; a stable latent form with an internalized and intact RCL within the protein core; and a cleaved form with a disrupted RCL [33]. Based on its structure characteristics, Serpin E1 interacts with uPA/tPA, vitronectin, and lipoprotein receptor through distinct domains [20]. The interaction between Serpin E1 and vitronectin stabilizes the active conformation of Serpin E1 by slowing the conversion of active to latent form, thereby modulating vitronectin and plasmin activity for ECM remodeling and promotion of angiogenesis in vivo [34]. However, in vitro experiments lacking ECM have shown that *H. pylori* infection, fibroblast- or GC cell-derived Serpin E1, and recSerpin E1 exert chemotactic and pro-proliferative effects on HUVECs, as well as stimulating tube formation of HUVECs in the present study. The findings suggest the involvement of alternative mechanism in Serpin E1-induced angiogenesis.

VEGFA serves as a key regulator of tumor angiogenesis, exerting precise control over the migration, proliferation, and vascular permeability of vascular endothelial cells. In malignancies, it is synthesized by diverse types of cells such as tumor and stromal cells like endothelial cells [35]. Hypoxia-inducible factor 1α and inflammatory cytokines like TNFα and interleukins have been demonstrated to induce VEGFA transcription [36]. Here, we found that Serpin E1, a new inflammatory cytokine secreted by fibroblasts and GC cells upon *H. pylori* infection, enhanced VEGFA expression and secretion, as well as tube information in HUVECs. This result was further supported by treating HUVECs with recSerpin E1. Moreover, blocking Serpin E1 using an anti-Serpin E1 antibody effectively suppressed recSerpin E1-mediated increases in VEGFA expression and tube formation in HUVECs. Furthermore, tumorigenicity assays on nude mice revealed that fibroblast-derived Serpin E1 promoted angiogenesis and/or VEGFA expression in subcutaneous and peritoneal tumors. Our previous study also unveiled an increased angiogenesis formation in the subcutaneous tumor of nude mice injected with GC cells overexpressing Serpin E1 [16]. Interestingly, Serpin E1 also stimulates VEGFA expression in CAFs in vitro, suggesting that both HUVECs and CAFs may serve as the primary sources of VEGFA within the gastric tumor microenvironment infected by *H. pylori*. MAPKs constitute a vast family of serine/threonine kinases, with ERK, p38, and JNK-mediated pathways playing a pivotal role. Upon receiving stimuli such as reactive oxygen species induced by *H. pylori* infection, it initiates a phosphorylation cascade of MAPKs, resulting in multiple cellular responses encompassing cell proliferation, apoptosis, invasion, metastasis, autophagy, et al. These responses are linked to the malignant behavers of tumor cells [37]. The activation of the p38 MAPK pathway induced by *H. pylori* infection in MKN45 cells has been shown to upregulate VEGFA expression through the use of its specific inhibitor SB203580 [38]. In liver cancer HepG2 cells, Panahi et al. [39] revealed that high glucose-induced inflammatory responses led to Serpin E1 upregulation, accompanied by activation of three crucial MAPK pathways. Therefore, we co-treat HUVECs with recSerpin E1 and (E)-osmundacetone (a universal inhibitor of MAPK phosphorylation), resulting in a significant reduction only in p38 MAPK phosphorylation levels accompanied by downregulation of VEGFA. This finding suggests that Serpin E1 facilitates VEGFA expression by activating the p38 MAPK signaling pathway in HUVECs. Therefore, Serpin E1 derived from fibroblasts and subsequently cancer cells stimulates VEGFA expression through the p38 MAPK pathway in HUVECs, thereby contributing to GC development and progression.

Additionally, we observed that Serpin E1 at a concentration of 10 ng/mL, below the normal plasma levels of 5–50 ng/ml, exhibited inhibitory rather than promotional effects on the in vitro growth of GC cells. Conversely, Fang's study used 10 ng/mL recSerpin E1 (obtained from the same company) to stimulate colony formation and migration of breast cancer SKBR-3 cells but did not provide information regarding seeded cell density [40]. The underlying reason for this discrepancy remained unclear. We speculated that the use of 96-well plates in our experiment and a relatively low cell density (0.5 × 10^4^) seeded in the culture plates may result in cytotoxicity and subsequent growth inhibition at a concentration of 10 ng/ml recSerpin E1. Furthermore, the specific type of GC cells used in our study might also contribute to the observed cytotoxicity at this concentration.

## Conclusions

This study elucidates a novel mechanism through which *H. pylori* infection promotes GC tumorigenesis and progression: *H. pylori* infection induces fibroblasts, especially CAFs, to upregulate Serpin E1 expression, subsequently triggering its expression in cancer cells by reciprocal interaction. The Serpin E1 derived from these two cell types then stimulates the migration and angiogenesis of HUVECs by activating p38 MAPK-mediated VEGFA expression, ultimately facilitating GC tumorigenesis and progression. Targeting Serpin E1 signaling represents a potential therapy choice for *H. pylori*-induced GC.

### Supplementary Information

**Additional file 1:** **Figure S1.** Identification of Serpin E1 expression in Serpin E1-knocked down and -overexpressed cancer-association fibroblasts (CAFs) by RT-qPCR and western blotting. ^*^P < 0.05; ^**^P *< *0.01; ^***^P < 0.001. **Figure S2.** Knockdown and overexpression of Serpin E1 impact the growth and apoptosis of CAFs. **A**, **C** Knockdown (**A**) or overexpression (**C**) of Serpin E1 inhibits or increases the viability of CAFs by CCK8 assay. **B**, **D** Knockdown (**B**) or overexpression (**D**) of Serpin E1 promotes apoptosis or protects against As_2_O_3_-induced apoptosis in CAFs by flow cytometry. Line and bar graphs show the mean ± SD; *p < 0.05, **p < 0.01, and ***p < 0.001. **Figure S3.** Identification of Serpin E1 protein expression in Serpin E1-overexpressed Hs738 cells (a human gastric fibroblast cell line). **Figure S4.** DAPI fluorescence intensity in Figure 5 was quantified in the subcutaneous (**A**) and peritoneal (**B**) tumors using Image J software. Data was presented as the mean ± SD (n=3), with “ns” indicating no statistically significant difference. **Figure S5.** Fibroblast-derived Serpin E1promptes the cycle progression of HUVECs and VEGFA expression in CAFs. **A** Cell-cycle distribution was analyzed by flow cytometry in HUVECs co-cultured with Serpin E1-overexpressed Hs738 cells. **B** Quantification of cell-cycle distribution. **C** Western blotting analysis of VEGFA in Serpin E1 knockdown and overexpression CAFs. Data were presented as the mean ± SD; ***p<0.001. **Table S1.** Primary and secondary antibodies used in the study.

## Data Availability

All data generated during this work is located within the article and Additional file. Upon reasonable request, more information is available from the corresponding author.

## Abbreviations

CAFs: Cancer-associated fibroblasts
CXCL1: C-X-C motif chemokine ligand 1
ELISA: Enzyme-linked immunosorbent assay
GC: Gastric cancer
ECM: Extracellular matrix
EGFP: Enhanced green fluorescent protein
*H. pylori*: *Helicobacter pylori*
HUVECs: Human umbilical vein endothelial cells
IF: Immunofluorescence
IHC: Immunohistochemistry
IL: Interleukin
MAPK: Mitogen-activated protein kinase
MIF: Macrophage migration inhibitory factor
PAI-1: Plasminogen activator inhibitor-type I
recSerpin E: Recombinant human Serpin E1
Serpin E1: Serpin family E member 1
TME: Tumor microenvironment
TNF: Tumor necrosis factor
tPA: Tissue-type plasminogen activator
uPA: Urokinase-type plasminogen activator
VEGFA: Vascular endothelial growth factor
