# Supplementary material for: Helicobacter pylori-induced fibroblast-derived Serpin E1 promotes gastric cancer growth and peritoneal dissemination through p38 MAPK/VEGFA-mediated angiogenesis
Source: Cancer Cell Int. 2023 Dec 16;23:326. doi: 10.1186/s12935-023-03177-1 (PMC10725580; doi:10.1186/s12935-023-03177-1)
Supplement: Supplementary file 1 — Additional file 1: Figure S1. Identification of Serpin E1 expression in Serpin E1-knocked down and -overexpressed cancer-association fibroblasts (CAFs) by RT-qPCR and western blotting. *P < 0.05; **P < 0.01; ***P < 0.001. Figure S2. Knockdown and overexpression of Serpin E1 impact the growth and apoptosis of CAFs. A, C Knockdown (A) or overexpression (C) of Serpin E1 inhibits or increases the viability of CAFs by CCK8 assay. B, D Knockdown (B) or overexpression (D) of Serpin E1 promotes apoptosis or protects against As2O3-induced apoptosis in CAFs by flow cytometry. Line and bar graphs show the mean ± SD; *p < 0.05, **p < 0.01, and ***p < 0.001. Figure S3. Identification of Serpin E1 protein expression in Serpin E1-overexpressed Hs738 cells (a human gastric fibroblast cell line). Figure S4. DAPI fluorescence intensity in Figure 5 was quantified in the subcutaneous (A) and peritoneal (B) tumors using Image J software. Data was presented as the mean ± SD (n=3), with “ns” indicating no statistically significant difference. Figure S5. Fibroblast-derived Serpin E1promptes the cycle progression of HUVECs and VEGFA expression in CAFs. A Cell-cycle distribution was analyzed by flow cytometry in HUVECs co-cultured with Serpin E1-overexpressed Hs738 cells. B Quantification of cell-cycle distribution. C Western blotting analysis of VEGFA in Serpin E1 knockdown and overexpression CAFs. Data were presented as the mean ± SD; ***p<0.001. Table S1. Primary and secondary antibodies used in the study. [file 12935_2023_3177_MOESM1_ESM.pdf]

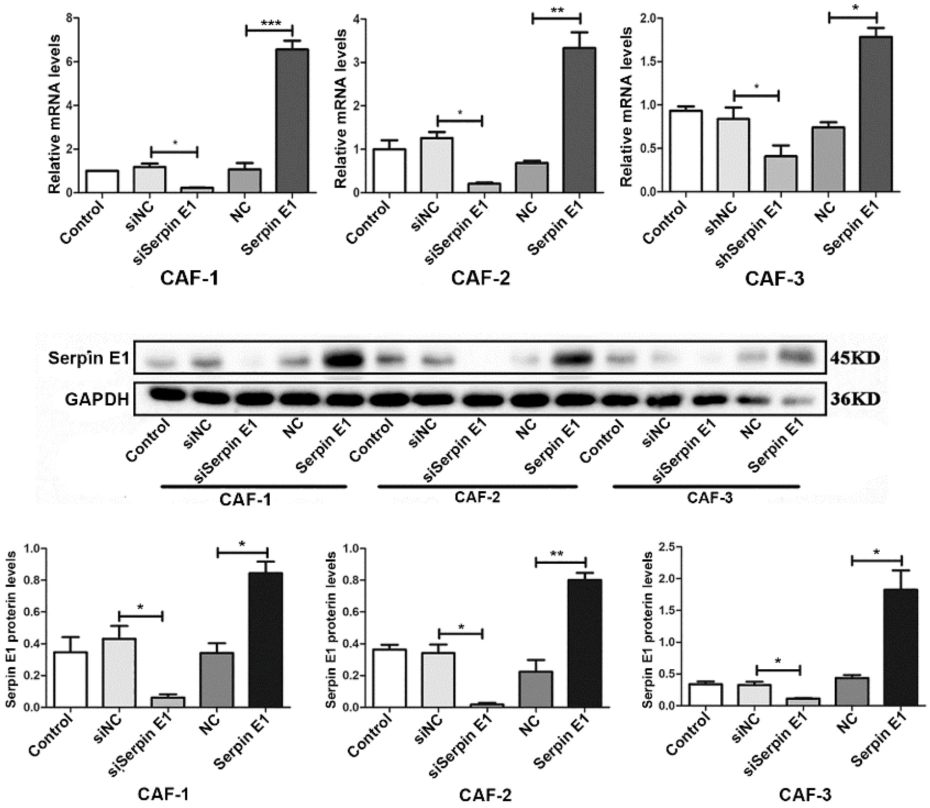

Figure S1. Identification of Serpin E1 expression in Serpin E1-knocked down and -overexpressed cancer-association fibroblasts (CAFs) by RT-qPCR and western blotting. $^{*}p$<0.05, $^{**}p$<0.01, and $^{***}p$<0.001

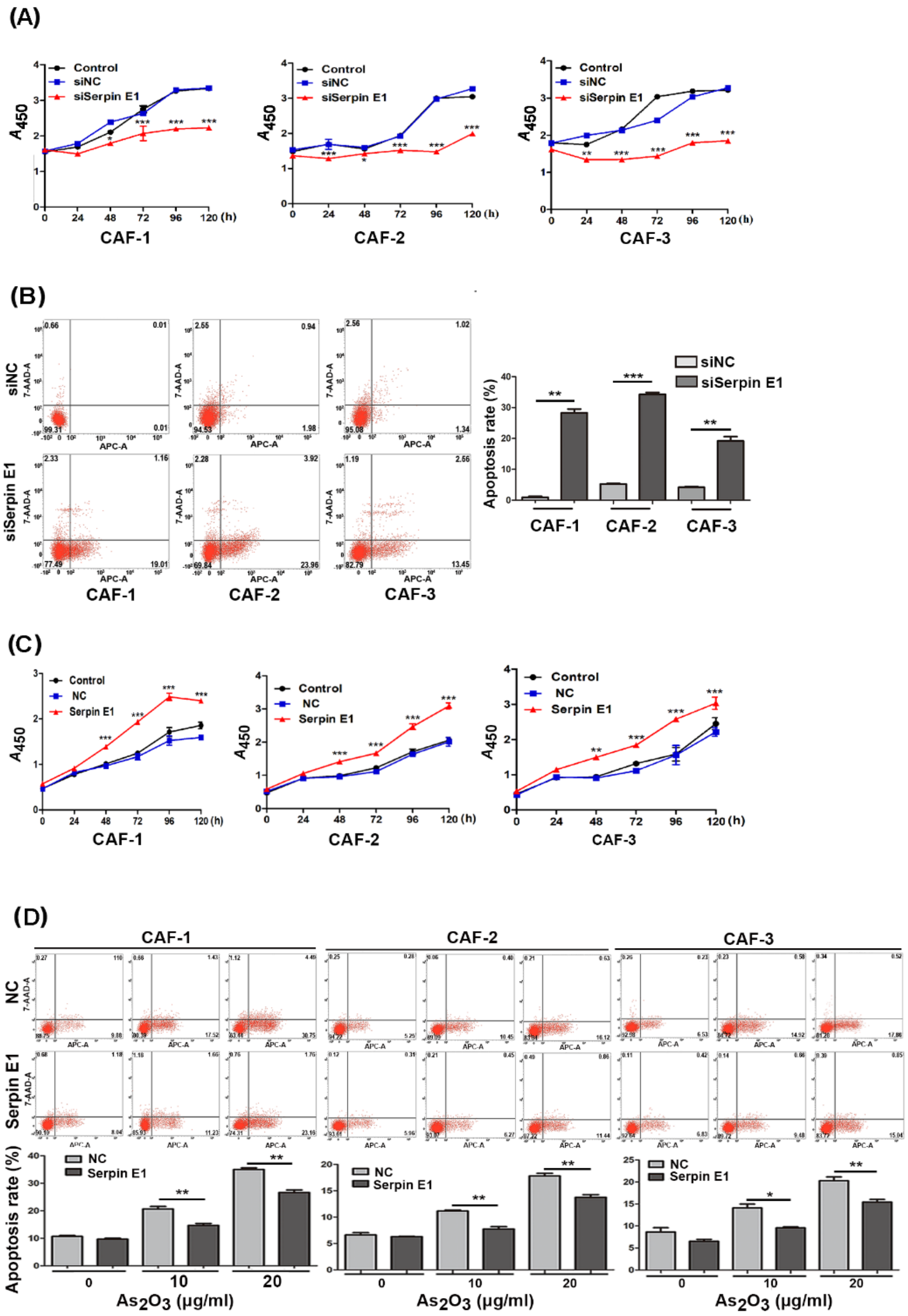

Figure S2. Knockdown and overexpression of Serpin E1 impact the growth and apoptosis of CAFs. **A, C** Knockdown (**A**) or overexpression (**C**) of Serpin E1 inhibits or increases the viability of CAFs by CCK8 assay. **B, D** Knockdown (**B**) or overexpression (**D**) of Serpin E1 promotes apoptosis or protects against As_2O_3-induced apoptosis in CAFs by flow cytometry. Line and bar graphs show the mean ± SD; *p<0.05, **p<0.01, and ***p<0.001

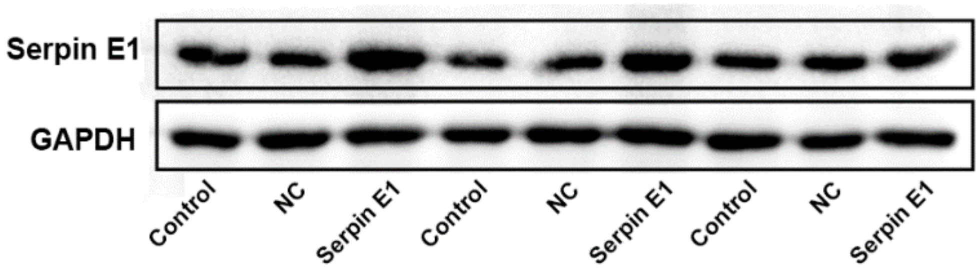

Figure S3. Identification of Serpin E1 protein expression in Serpin E1-overexpressed Hs738 cells (a human gastric fibroblast cell line)

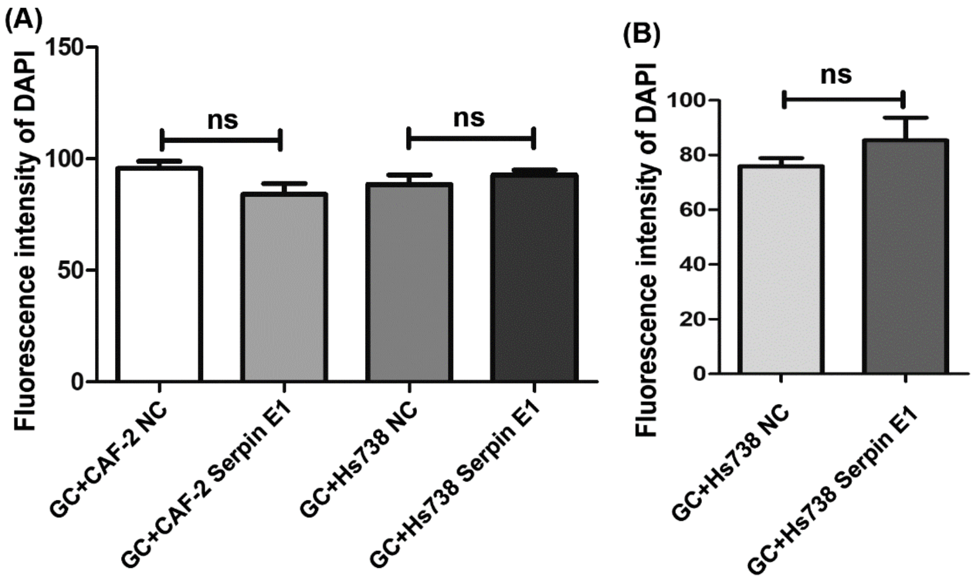

Figure S4. DAPI fluorescence intensity in Figure 5 was quantified in the subcutaneous (**A**) and peritoneal (**B**) tumors using Image J software. Data was presented as the mean ± SD (n=3), with "ns" indicating no statistically significant difference.

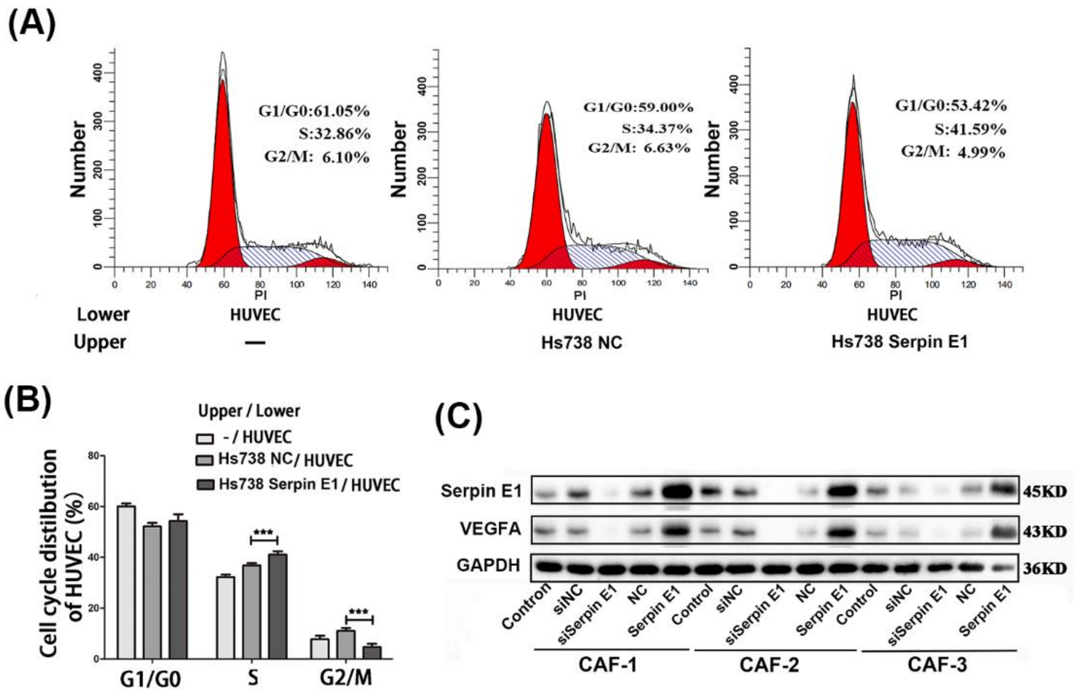

Figure S5. Fibroblast-derived Serpin E1promptes the cycle progression of HUVECs and VEGFA expression in CSFs. **A** Cell-cycle distribution was analyzed by flow cytometry in HUVECs co-cultured with Serpin E1-overexpressed Hs738 cells. **B** Quantification of cell-cycle distribution. **C** Western blotting analysis of VEGFA in Serpin E1 knockdown and overexpression CAFs. Data were presented as the mean ± SD; ****p*<0.001.

Table S1 Primary and secondary antibodies used in the study

Proteins	Antibodies	Dilution (Western blot)	Dilution (IHC/IF)	Catalog number	Company
Serpin E1	Rabbit pAb	1:800	1:200	Ab66705	Abcam
Ki67	Mouse mAb	NA	1:200	9449S	CST
CD31	Rabbit mAb	NA	1:250	Ab182981	Abcam
VEGFA	Rabbit mAb	1:600	1:250	EP1176Y	Abcam
P-p38	Rabbit pAb	1:1000	NA	28796-1-AP	Proteintech
p38	Mouse mAb	1:1000	NA	66234-1	Proteintech
P-ERK1/2	Rabbit mAb	1:1000	NA	27833-1-AP	Proteintech
ERK1/2	Rabbit mAb	1:1000	NA	11257-1-AP	Proteintech
P-JNK	Rabbit pAb	1:2000	NA	80024-1-RR	Proteintech
JNK	Mouse mAb	1:1000	NA	66210-1	Proteintech
CK18	Mouse pAb	NA	1:400	66187-1-Ig	Proteintech
GAPDH	Rabbit pAb	1:1000	NA	10494-1-AP	Proteintech
Secondary antibody	Gost IgG (HRP -conjugated)	1:8000	1:400	SA00001-2	Proteintech
Secondary antibody	Gost IgG (CoraLite594 -conjugated)	NA	1:300	SA00013-4	Proteintech
